# MicroRNA-9 regulates the development of knee osteoarthritis through the NF-kappaB1 pathway in chondrocytes

**DOI:** 10.1097/MD.0000000000004315

**Published:** 2016-09-09

**Authors:** Ronghe Gu, Ning Liu, Simin Luo, Weiguo Huang, Zhengang Zha, Jie Yang

**Affiliations:** a Department of Orthopedics, The First Affiliated Hospital of Jinan University, Guangzhou; b Department of Orthopedics, The First People's Hospital of Nanning, Nanning, China.

**Keywords:** chondrocyte, IL-6, knee osteoarthritis, miR-9, MMP-13, NF-kappaB1 pathway

## Abstract

It has been suggested that microRNA-9 (miR-9) is associated with the development of knee osteoarthritis (OA). This study was aimed to investigate the association between the mechanism of miR-9 targeting nuclear factor kappa-B1 (NF-κB1) and the proliferation and apoptosis of knee OA chondrocytes.

Cartilage samples were collected from 25 patients with knee OA and 10 traumatic amputees, and another 15 OA rat models, together with 15 rats without knee OA lesions were also established. MiR-9 expressions in both knee OA cartilage and normal cartilage samples were detected using quantitative real-time PCR. The expressions of related genes (*NF-κB1*, *IL-6*, and *MMP-13*) in the two groups were also detected. Dual luciferase reporter gene assay was employed to examine the effect of miR-9 on the luciferase activity of NF-κB1 3′UTR. Knee OA chondrocytes were transfected with miR-9 mimics, miR-9 inhibitor, and NF-κB1 siRNA, respectively, and changes in cellular proliferation and apoptosis were detected via MTT assay and flow cytometric analysis, respectively. Western blotting assay was used to detect the expressions of NF-κB1, interleukin-6 (IL-6), and matrix metalloproteinase-13 (MMP-13).

According to results from human OA samples and rat OA models, miR-9 was significantly downregulated in knee OA cartilage tissues compared with normal cartilage tissues (*P* < 0.01). The expressions of NF-κB1, IL-6, and MMP-13 in knee OA cartilage tissues were significantly higher than those in normal cartilage tissues (*P* < 0.01). Dual luciferase reporter gene assay showed that miR-9 could bind to the 3′UTR of NF-κB1 and significantly inhibit the luciferase activity by 37% (*P* < 0.01). Upregulation of miR-9 or downregulation of NF-κB1 could promote cell proliferation and suppress cell apoptosis.

Conclusively, downregulated miR-9 can facilitate proliferation and antiapoptosis of knee OA chondrocytes by directly binding to NF-kB1, implying that stimulating miR-9 expressions might assist in treatment of knee OA.

## Abstract 2

Supplemental Digital Content is available in the text

## 1 Introduction

Osteoarthritis (OA) is classified as a degenerative disease that affects both cartilage and its adjacent issues. Knee OA is the most common type of OA^[1]^ and it is considered as a chronic joint disease characterized by knee cartilage degeneration, damage, and osteoarthritis.^[2]^ The incidence of knee OA is frequent (50%) among the population aged above 60 years, and peaks (80%) at 75 years.^[3]^ Knee OA may gradually lead to patients’ functional loss, which is mainly manifested as joint pain, swelling, stiffness, joint effusion, and movement disorder.^[2]^ According to former epidemiological investigations, the etiology of knee OA is probably associated with several risk factors, including genetics, cartilage metabolism, inflammation, and immunity.^[4]^ Based on the pathogenesis of knee OA, current exploration of knee OA therapies are principally concerned with researches on endogenous hormones, oxygen radicals, calcified layer structure of cartilage, autoimmunity, and cytokines.^[3]^ However, few researches have been able to figure out the exact molecular mechanism underlying knee OA development and progression.^[5–7]^

Certain noncoding RNA molecules (microRNAs), such as miR-9, miR-22 (Gene ID: 407004), and miR-146 (Gene ID: 406938), have been reported to modify target gene expressions by targeting their mRNA 3′untranslated regions (UTR).^[8]^ Interestingly, Song and his colleagues^[9]^ found that miR-9 expressions were significantly decreased in OA chondrocytes in comparison to normal ones, and chondrocytes’ apoptosis was thereby regulated due to miR-9's targeting protein (PRTG, Gene ID: 283659). However, one expression profiling asserted that miR-9 was upregulated in OA cartilages and OA bones.^[10]^ The controversy enabled us to further explore inherent correlations between miR-9 and OA chondrocytes. In addition, it was demonstrated that miR-9 could suppress proliferation and invasion of diverse cancer cells (e.g., nasopharyngeal carcinoma, ovarian cancer, and gastric cancer), by binding to corresponding genes, such as *C-X-C motif chemokine receptor 4* (*CXCR4*, Gene ID: 7852), *talin 1* (*TLN1*, Gene ID: 7094), and *nuclear factor kappa-B1* (*NF-κB1*, Gene ID: 4790).^[11–14]^

Among the aforementioned genes, *NF-κB1* needs to be emphasized, since the activation of NF-κB signaling pathways would trigger release of pro-inflammatory cytokines that play a pivotal role in altering the degree of cartilage damage and the balance of bone metabolism, including interleukin-1β (IL-1β) and tumor necrosis factor-alpha (TNF-α, Gene ID: 7124).^[15,16]^ It was suggested that IL-1β might induce upregulation of matrix metalloproteinase-3 (MMP-3, Gene ID: 4314) and MMP-13 (Gene ID: 4322), which are vital elements that could contribute to the irreversible destruction of cartilage matrix.^[17]^ In turn, interaction of high-mobility protein groups B1 (HMGB1, Gene ID: 3146) and IL-1β enhanced the transcriptional activity of NF-κB, and expressional levels of interleukin-6 (IL-6, Gene ID: 3569), IL-8, chemokine C-C motif ligand 2 (CCL2, Gene ID: 6347), CCL20 (Gene ID: 6364), and even MMPs were changed. The complicated bioreactions would possibly result in the induction of synovitis and joint destruction.^[18]^ Furthermore, certain components, such as interleukin-1 (IL-1), TNF, and lipopolysaccharide (LPS), could specifically stimulate chondrocytes to fortify expressions of epithelium-specific Ets transcription factor 1 (ESE-1, Gene ID: 1999) through modifying NF-κB1and p65 (NF-κB2, Gene ID: 5970).^[19,20]^ All in all, NF-κB1 appears to run through the development metabolism of knee OA.^[21–23]^

Nonetheless, so far deficient studies can explain clearly how miR-9 regulates NF-κB1 and whether the regulation could influence development of knee OA. Therefore, the present study was designed to systematically clarify the potential correlations of miR-9/NF-κB1 and knee OA development, which may be conducive to exploitation of novel diagnostic and therapeutic strategies for knee OA.

## 2 Materials and methods

### 2.1 Patients and tissue samples

Human knee OA cartilage samples were collected from patients (n = 25; 15 females and10 males; age 54–78 years) who underwent total knee replacement operations in our hospital (Supplementary Table 1). All patients were diagnosed according to the American College of Rheumatology criteria^[24]^ and evaluated by a certified rheumatologist. Matched normal cartilage tissues were obtained from traumatic amputees admitted in our department with no history of joint pain (n = 10; 4 males and 6 females; age 50–73 years). Tissue samples were frozen in liquid nitrogen immediately after surgery and were stored at −80°C until usage. This research was approved by the Research Ethics Committee of The First Affiliated Hospital, Jinan University and The First People's Hospital of Nanning and all participants signed the informed consents.

### 2.2 Establishment of OA rat models

Thirty 8-week male Sprague–Dawley (SD) rats (weight: 250–300 g) were randomly allocated into normal group (n = 15) and knee OA group (n = 15). The normal group with no knee lesions was not treated, while knee OA group was managed with cutoff of anterior cruciate ligament and excision of medial meniscus in line with Hulth method.^[25]^ After surgery, each rat was injected with amikacin (specification: 100,000 U/mL/d; concentration: 10 mg/kg) for consecutive 4 days. All the rats were executed 4 weeks after surgery and their knee joints were simultaneously taken out. The animal experiments accorded with the National Institutes of Health Guidelines for Care and Usage of Laboratory Animals and were approved by the Ethics Committee of Experimental Animal Center, Shandong Province.

### 2.3 RNA extraction and RT-PCR

Total RNA extraction from human knee OA cartilage tissues, normal cartilage tissues, and chondrocytes was conducted using TRIzol reagent kit (Invitrogen, Carlsbad, CA, USA) based on the instruction. Complementary DNA (cDNA) was acquired using the Omniscript reverse transcription kit (Qiagen, Germany). Real-time quantitative RT-PCR assay was conducted using the ABI7500 quantitative PCR instrument (Applied Biosystems, Foster City, CA, USA) in order to detect the relative expression levels of miR-9 and mRNA of NF-κB1, IL-6, and MMP-13. The primers of miR-9, NF-κB1, IL-6, and MMP-13 (purchased from Invitrogen) were used as the followings: miR-9 forward, 5′- CGGGGTTGGTTGTTATCTTTGG-3′ and reverse 5′-GCTTTATGAAGACTCCACACCAC-3′; NF-κB1 sense 5′-ACAGCAGATGGCCCATACCT-3′ and antisense 5′-CATACATAACGGAAACGAAATCCTCT-3′; IL-6 sense, 5′-CAATGAGGAGACTTGCCTGG -3′ and antisense 5′-GCACAGCTCTGGCTTGTTCC-3′; MMP-13 forward 5′-CCCCAACCCTAA ACATCCAA-3′ and reverse 5′-AACAGCTCCGCATCAACCT-3′. The relative expression level of miR-9 and mRNA of p50, IL-6, and MMP-13 were calculated using the 2^−ΔΔCt^ method.^[26]^ In particular, we determined the Ct values of all studied samples to calculate ΔCt, which equaled the difference between Ct-value of target mRNAs (miR-9 or mRNAs of NF-κB1, IL-6, and MMP-13) and that of U6 snRNA. The ΔΔCt value was derived from the difference between ΔCt in the experimental group and the control group. Thus, 2^−ΔΔCt^ was considered equal to the fold of expressions of miR-9 or NF-κB1 RNA or IL-6 RNA or MMP-13 RNA and those of U6 snRNA.^[27,28]^ All the above assays were replicated for 3 times.

### 2.4 Cell culture

Chondrocytes were extracted from knee OA cartilage samples as previously described.^[29]^ Cells were seeded at the density of 1.5 × 10^4^ cells/cm^2^ and cultured in Dulbecco's Modified Eagles Medium (DMEM; Gibco) containing10% heat-inactivated fetal calf serum (FCS; Gibco), streptomycin (100 mg/mL; Gibco,Grand Island, NY, USA), and penicillin (100 units/mL; Gibco) in incubator at 37°C with 5% CO_2_. The medium was replaced every 2 to 3 days and cultured chondrocytes between the second and third passage were used in the experiments.

### 2.5 Cell transfection

Chondrocytes were divided into 4 different groups, including the scramble group, miR-9 mimics group, miR-9 inhibitor group, and NF-κB1 siRNA group. They were transfected with scramble miRNA mimics as the negative control, miR-9 mimics, miR-9 inhibitor, and NF-κB1siRNA, respectively (purchased from Gene Pharma, Shanghai, China). Cells were transfected by Lipofectamine 2000 (Invitrogen) and cultured in incubator at 37°C with 5% CO_2_. Cell culture continued after the complete medium was replaced after 6 to 8 hours.

### 2.6 MiRNA target prediction and dual-luciferase reporter assay

MiRNAs targets were predicted using the Target Scan (https://www.targetscan.org). The wild-type and mutant-type of NF-κB1 3′UTR luciferase reporter vectors were constructed. MiR-9 mimics or scramble were cotransfected with the constructed wild-type or mutant-type luciferase reporter vector into chondrocytes using Lipofectamine 2000 (Invitrogen). The pRL-TK control vector (Promega, Madison, WI, USA) was transfected and served as the control. The luciferase activity was analyzed with the Dual-Luciferase Reporter Assay System (E1910; Promega) after cells were transfected for 48 hours.

### 2.7 MTT assay

MTT [3-(4, 5-dimethylthiazol-2-yl)-2, 5-diphenyl-tetrazolium bromide] assays were used to evaluate the proliferation of chondrocytes. Briefly, transfected chondrocytes were cleaned twice by phosphate buffered solution and then were cultured to the density of 80%. Cells were digested into cell suspensions with trypsin and then cells were counted using cell counter. Chondrocytes were inoculated into 96-well plates with 3 × 10^3^ to 6 × 10^3^ cells/well and a total of 6 wells were repeated. DMEM medium was added as a zero well in unseeded cell wells. Cells were detected after they were transfected for 24, 48, 72, and 96 hours, respectively. MTT (20 μL, 5 mg/mL, Sigma, St. Louis, MO, USA) was added into each well and cell culture was sustained for 4 hours at 37°C in incubator with 5% CO_2_. Subsequently, dimethyl sulfoxide (DMSO, 150 μL) was added into each well and cells were lightly shaken for 10 minutes in order to dissolve the crystals. Samples were detected using a microplate reader (SpectraMAX Plus; Molecular Devices, Sunnyvale, CA) at a wavelength of 490 nm. MTT curve was drawn in which the absorbance value is represented by the vertical axis and the transfection time is represented by the horizontal axis.

### 2.8 Flow cytometric analysis

Annexin V-fluorescein isothiocyanate (Annexin V-FITC) and propidium iodide apoptosis detection kit (Becton Dickinson, Franklin Lakes, NJ, USA) were used to evaluate the apoptosis of chondrocytes. In brief, chondrocytes were washed twice with cold phosphate buffered solution after 48-hour transfection. Then, cells were resuspended with binding buffer to reach a density of 0.5 to 1 × 10^6^/mL. This suspension (100 μL) was incubated with 5 μL of Annexin V-FITC and propidium iodide for 15 minutes in the dark at room temperature. After adding 400 μL binding buffer to each tube, cells were analyzed by flow cytometry (Beckman FC 500 MCL/MPL).

### 2.9 Detection of caspase-3 activity

After cells were transfected for 48 hours, the caspase-3 activity was detected using the Caspase Colorimetric Assay Kit (KeyGEN, Nanjing, China). Cells were lysed in lysis buffer on ice for 20 minutes for detecting the activity of caspase-3. Supernatants after centrifugation were incubated with the caspase substrate at 37°C in the reaction buffer for 4 hours. Samples were detected using a microplate reader (Spectra MAX Plus, Molecular Devices) at the wavelength of 405 nm. The relative caspase-3 activity was calculated as the percentage of A405 values in the treatment group over that in the control group.

### 2.10 Western blotting assay

The expression level of NF-κB1, IL-6, and MMP-13 were detected by western blotting assay. Cellular proteins were extracted after 48-hour transfection. BCA method was used to evaluate the protein density. Equal amount of proteins for each group were loaded and separated by sodium dodecyl sulfate-polyacrylamide gel electrophoresis (SDS-PAGE), transferred onto polyvinylidene fluoride membranes and blocked with 5% skim milk. Membranes were incubated at 4°C overnight with primary antibody (NF-κB1, IL-6, and MMP-13) and glyceraldehyde-3-phosphate dehydrogenase (GAPDH) antibody (CST, American), respectively. Membranes were washed three times using tris buffered saline tween (TBST, 10 minutes each time) and incubated with horseradish-peroxidase-linked secondary antibodies for 1 hour at room temperature. Membranes were washed again with TBST for another three times (10 minutes each time) and signal detection was performed using the Super ECL Plus Detection Reagent (Applygen Technologies Inc., Beijing, China).

### 2.11 Statistical analysis

All statistical analyses were performed by SPSS 19.0 software (SPSS, Inc., Armonk, NY, USA) (IBM Corp., Armonk, NY). Significant differences of numerical data which were represented in the form of mean ± SD among ≥3 groups were estimated using the analysis of variance. Difference between two groups was analyzed by the unpaired *t*-tests. *P* < 0.05 was considered as statistically significant.

## 3 Results

### 3.1 MiR-9 and related genes expression in knee OA clinical specimens and knee OA rat models

**Figure 1:**
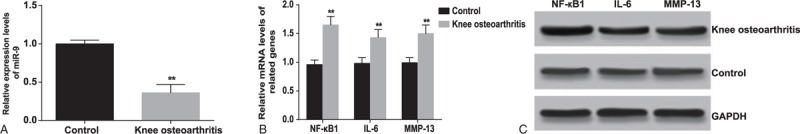
The relative expressions of miR-9 and related genes (*NF-κB1*, *IL-6*, and *MMP-13*) detected in human knee OA and normal cartilage tissues. (A) The miR-9 expressions were detected with quantitative real-time PCR. (B) The relative mRNA levels of *NF-κB1*, *IL-6*, and *MMP-13* were detected by quantitative real-time PCR. (C) The relative protein levels of *NF-κB1*, *IL-6*, and *MMP-13* were detected by western blot assay. The results were from 3 independent experiments. The data are presented as mean ± SD. ∗∗*P* < 0.01, compared with the control group.

**Figure 2:**
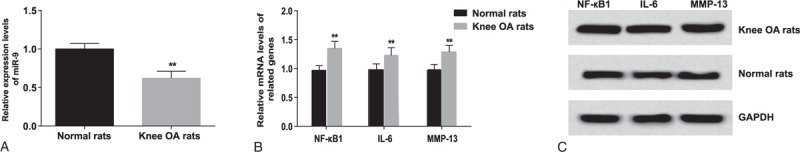
The relative expressions of miR-9 and related genes (*NF-κB1*, *IL-6*, and *MMP-13*) detected in knee OA and normal cartilage tissues of rat models. (A) The miR-9 expressions were detected with quantitative real-time PCR. (B) The relative mRNA levels of *NF-κB1*, *IL-6*, and *MMP-13* were detected by quantitative real-time PCR. (C) The relative protein levels of *NF-κB1*, *IL-6*, and *MMP-13* were detected by western blot assay. The results were from 3 independent experiments. The data are presented as mean ± SD. ∗∗*P* < 0.01, compared with the control group.

Quantitative real-time PCR was used to evaluate the expression level of miR-9 in 25 knee OA cartilage tissues and 10 normal cartilage tissues (Fig. 1A). The expression of miR-9 in knee OA cartilage tissues was significantly lower than that in normal tissues (*P* < 0.01). The mRNA and protein expression of related genes (NF-κB1, IL-6, and MMP-13) in 25 knee OA cartilage tissues and 10 normal cartilage tissues were also examined. Both mRNA and protein expression levels of NF-κB1, IL-6, and MMP-13 in knee OA cartilage tissues were significantly higher than those in normal tissues (*P* < 0.01) (Fig. 1B, C). Subsequently, assessment of rat models also demonstrated reduced miR-9 expressions (Fig. 2A) as well as increased NF-κB1, IL-6, and MMP-13 expressions (Fig. 2B, C) in OA rats’ cartilage tissues when compared with normal rats’ cartilage tissues.

### 3.2 Targeting NF-κB1 by miR-9

**Figure 3:**
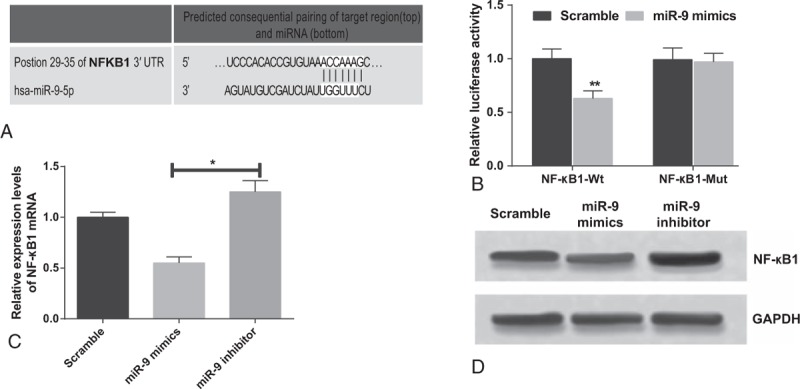
NF-κB1is a target gene of miR-9. (A) Binding of miR-9 to NF-κB1 3′-UTR predicted by Target Scan. (B) Dual luciferase reporter gene assay revealed that miR-9 significantly decreased the luciferase activity of NF-κB1 wild-type (wt) 3′UTR. (C) NF-κB1 mRNA levels in chondrocytes transfected with miR-9 mimics, miR-9 inhibitors, or scramble sequence were examined by qRT-PCR. (D) NF-κB1 protein levels were detected by western blotting using glyceraldehyde-3-phosphate dehydrogenase (GAPDH) as a loading control. The results were from 3 independent experiments. The data are presented as the mean ± SD. ∗∗*P* < 0.01 versus corresponding control; ∗*P* < 0.05 versus corresponding control.

A putative conserved binding site for miR-9 at nucleotide position 29–35 of human NF-κB13′UTR is predicted using the Target Scan. Perfect base pairing was observed between the seed sequence of mature miR-9 and the 3′UTR of NF-κB1 mRNA (Fig. 3A). Dual luciferase reporter gene assays revealed that miR-9 significantly decreased the luciferase activity of NF-κB1wild-type by 37% (*P* < 0.01), while it had no significant effect on the NF-κB1 mutant-type 3′UTR luciferase activity (Fig. 2B). Both RT-PCR and western blot assays showed that the expression levels of NF-κB1 mRNA and protein were significantly decreased in the miR-9 mimics group and increased in the miR-9 inhibitor group when compared with the scramble group (*P* < 0.05) (Fig. 3C, D).

### 3.3 MiR-9 targeted NF-κB1 to promote chondrocytes proliferation

**Figure 4:**
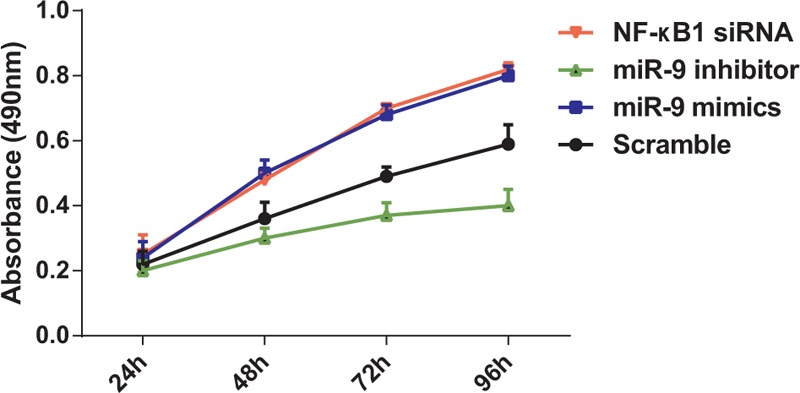
The proliferation of knee OA chondrocytes was increased by miR-9. The A490 values of cells were measured by MTT assay. The results (mean ± SD) were obtained from 6 independent experiments.

Compared with the scramble group, the proliferation of chondrocytes was significantly increased when cells were transfected with miR-9 mimics and NF-κB1 siRNA for 48 hours (all *P* < 0.05). This difference was more significant as the transfection time extended. However, miR-9 mimics or NF-κB1 siRNA did not have significant effect on promoting cell proliferation (*P* > 0.05). The proliferation of chondrocytes was significantly decreased after they were transfected with miR-9 inhibitor for 48 hours compared with the other three groups (all *P* < 0.05) (Fig. 4).

### 3.4 MiR-9 targeted NF-κB1 to inhibit cell apoptosis

**Figure 5:**
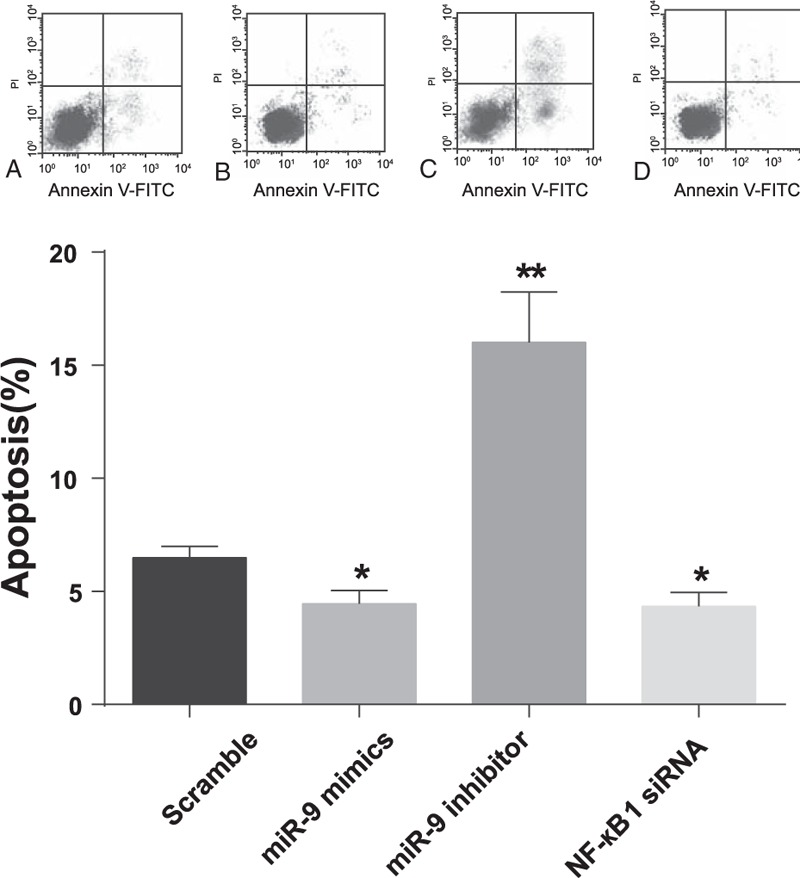
The apoptosis of chondrocytes was inhibited by miR-9. The apoptosis ability of chondrocytes at 48 hours after transfection was detected by the flow cytometric analysis. The results (mean ± SD) were from 6 independent experiments. ∗*P* < 0.05 versus scramble group; ∗∗*P* < 0.01 versus scramble group.

Results from flow cytometric analysis revealed that the apoptosis rate (mean ± SD) of cells transfected with miR-9 mimics and NF-κB1 siRNA were 4.46 ± 0.58% and 4.34 ± 0.62% without significant difference (*P* > 0.05). However, apoptosis rates in those cells were significantly lower compared with the scramble group (6.29 ± 0.50%) (*P* < 0.05). The apoptosis rate in the miR-9 inhibitor group was 16.01 ± 2.23%, which was significantly higher than those in the other three groups (all *P* < 0.01) (Fig. 5). These findings indicated that miR-9 could inhibit the apoptosis of chondrocytes and downregulation of NF-κB1 also could suppress the apoptosis of chondrocytes.

### 3.5 Caspase-3 activity

**Figure 6:**
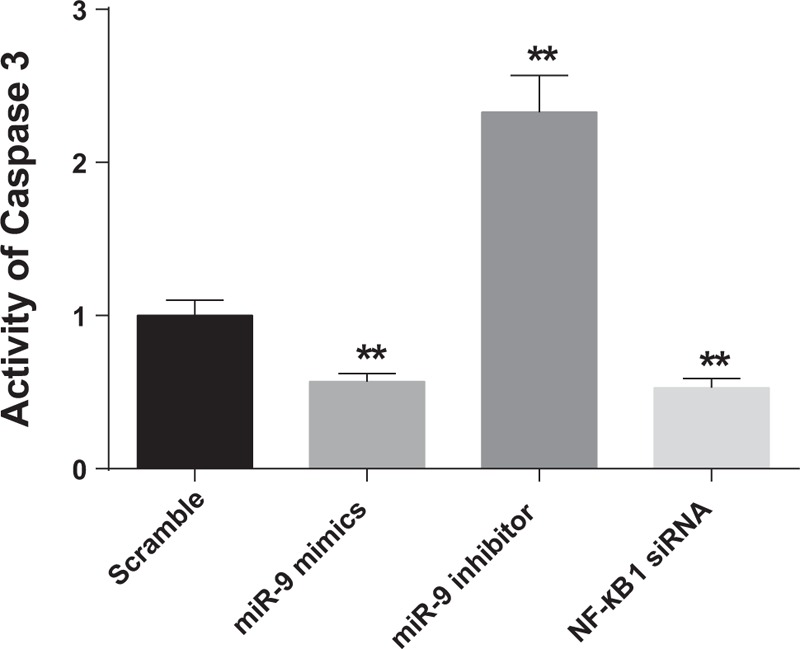
The analyzed caspase-3 activity in cells after 48-hour transfection with scramble sequence, miR-9 mimics, miR-9 inhibitor, or NF-κB1 siRNA. The results were from 6 independent experiments. The data were presented as the mean ± SD. ∗∗*P* < 0.01, compared with the scramble group.

The caspase-3 activity of cells transfected with miR-9 inhibitor was 2.33 ± 0.24, exhibiting significant difference when compared with those of the other three groups (all *P* < 0.01). The caspase-3 activities of cells transfected with miR-9 mimics and NF-κB1siRNA were 0.57 ± 0.05 and 0.53 ± 0.06 with no significant difference (*P* > 0.05), while they were significantly lower than that in the scramble group (1.00 ± 0.10) (*P* < 0.01) (Fig. 6).

### 3.6 MiR-9 regulated the NF-κB1 signaling pathway

**Figure 7:**
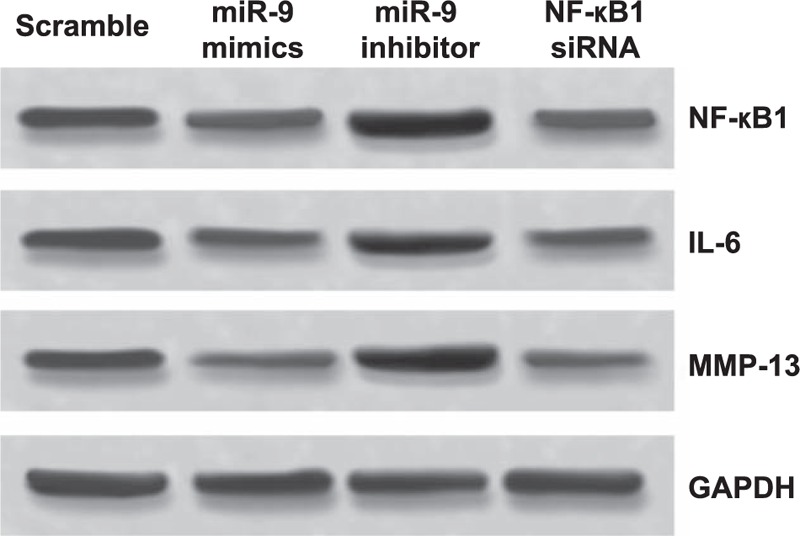
The effect of miR-9 on the NF-κB1 signaling pathway via the inhibition of NF-κB1. Western blot showed that miR-9 could suppress the NF-κB1 expression to decrease the NF-κB1 signaling pathway related proteins including IL-6 and MMP-13 in human knee OA chondrocytes.

The expression levels of NF-κB1 were significantly inhibited by miR-9 mimics and increased by miR-9 inhibitor. Besides that, the protein levels of IL-6 and MMP-13 were suppressed by miR-9 mimics and NF-κB1 siRNA, while they were promoted by miR-9 inhibitor (Fig. 7). Since the NF-κB1 signaling pathway is involved in the apoptosis and proliferation of tumor cells, we suspected that miR-9 may promote the proliferation of chondrocytes and suppress the apoptosis of human pituitary knee OA chondrocytes through regulating the NF-κB1 signaling pathway by targeting NF-κB1 and this is consistent with the results from the western blotting.

## 4 Discussion

Progression of knee OA would contribute to knee cartilage degeneration and damage which eventually triggers disability,^[30]^ yet few researchers could explain comprehensively the intrinsic mechanisms. In retrospect, such miRNAs as miR-9, miR-27 (Gene ID: 407018), miR-140 (Gene ID: 406932), and miR-146 have been indicated to be abnormally expressed in OA patients.^[31,32]^ Likewise, certain genes (e.g., *NF-κB1*, *IL-6*, and *MAP-13*) were also reported to be overexpressed in patients with knee OA, especially in the early stage of OA.^[33,34]^ Hence, the current study was aimed to build internal relations among miR-9, *NF-κB1*, *IL-6*, and *MAP-13* in knee OA cartilages.

The dual-luciferase reporter assay in this study displayed that NF-κB1 expressions were modulated by miR-9 in chondrocytes, which was consistent with results drawn from uveal melanoma cells, ovarian cancer cells, and gastric adenocarcinoma cells.^[14,35–37]^ Furthermore, our study discovered that miR-9 mimics suppressed the NF-κB1 protein expression level in knee OA chondrocytes and the downregulation of miR-9 could trigger an increase in NF-κB1 expressions occurred at both gene and transcription levels in chondrocytes. Hence, we concluded that the expression of NF-κB1 at both mRNA and protein levels were modulated by miR-9. Besides, the interaction between miR-9 and NF-κB1 was hypothesized to suppress apoptosis during chondrogenesis,^[9]^ since the caspase-3 experiments conducted in this study^[38]^ exhibited that higher caspase-3 levels along with increased apoptosis were observed in cells transfected with miR-9 inhibitors, whereas lower caspase-3 activity accompanied by decreased apoptosis were present in cells transfected with miR-9 mimics and p50 siRNA.

To elucidate effects of miR-9 and NF-κB1 on downstream molecules, expressions of IL-6 and MMP-13 were also compared between normal tissues and knee OA tissues. Previous studies showed that miR-9 modulated the secretion of MMP-13^[10]^ and that miR-9 was able to inhibit tumorigenesis by suppressing the activity of IL-6.^[39]^ In addition, NF-κB1, IL-6, and catabolic marker protein MMP-13, which is the matrix-degrading enzyme, were also overexpressed in patients with knee OA.^[40–42]^ Data in the present study indicated that both IL-6 and MMP-13 were overexpressed in knee OA cartilage tissues compared with normal cartilage tissues. Moreover, both IL-6 and MMP-13 were significantly decreased after chondrocytes were transfected with NF-κB1 siRNA, which was consistent with the trend observed in the miR-9 mimics group. Thus, this study displayed that reduced expressions of IL-6 and MMP-13 were attributed to regulated NF-κB1expressions targeted by miR-9.

Interestingly, multiple studies have documented that NF-kB could turn on genes that keep cells proliferating. For example, restrained NF-kB expressions were believed to impede proliferation of HeLa cells and upregulated NF-kB might indirectly lead to H-ras oncogene-induced cell proliferation.^[43,44]^ However, the current study reported that in knee OA tissues, upregulated NF-kB expressions because of inhibited miR-9 expressions were associated with increased knee OA cell proliferation. The difference might be explained by the distinctions of cell types and intercellular environment. In fact, miR-9 was documented to be lowly expressed in the knee OA chondrocytes and its low expression was correlated to increased chondrocyte apoptosis.^[9]^ Besides, miR-9 has been ascertained to negatively regulate NF-kB1 expressions, thereby indicating that downregulated miR-9 would accelerate NF-kB expressions and restrain cell proliferation.^[36,45]^ Furthermore, another assumption that could not be ignored was that NF-kB could indeed simultaneously control various genes of different functions (e.g., antiapoptosis and antiproliferative effects). And NF-kB might be able to facilitate more expressions of antiapoptosis genes than those of antiproliferative genes in chondrocytes, while the correlation was reversed in other cells. Thus, the expressional imbalance of genes within distinct cells needs to be further explored to verify our hypothesis.

Although the relationship between miR-9 and NF-κB1 with respect to knee OA formation has been demonstrated, this study has a small sample size which is the main limitation. Furthermore, as cellular experiments in this study were merely based on chondrocytes from the same donor, chondrocytes from diverse donors should also be attempted. In addition, more exploration of NF-κB1 pathway with aid of Ingenuity Pathway Analysis would make roles of miR-9 and NF-κB1 in development of knee OA more convincible. Finally, animal models with knockout of specific genes (e.g., miR-9 and NF-κB1) could also be the following research focus. All in all, the molecular mechanism of miR-9 and NF-κB1 pathway with respect to the formation and progression of knee OA should be further studied.

In conclusion, miR-9 exhibited significantly lower expressions in knee OA tissues when compared with normal tissues, while NF-κB1, IL-6, and MMP-13 expressions were relatively higher in knee OA tissues. The targeting of miR-9 to NF-κB1 may enhance proliferation and suppress apoptosis of knee OA chondrocytes through modification of IL-6 and MMP-13. As a result, miR-9 and NF-κB1 could potentially serve as diagnostic biomarkers and therapeutic targets for patients with knee OA.

## Supplementary Material

Supplemental Digital Content
