# Supplementary material for: MicroRNA-9 regulates the development of knee osteoarthritis through the NF-kappaB1 pathway in chondrocytes
Source: Medicine (Baltimore). 2016 Sep 9;95(36):e4315. doi: 10.1097/MD.0000000000004315 (PMC5023855; doi:10.1097/MD.0000000000004315)
Supplement: Supplemental Digital Content [file medi-95-e4315-s001.docx]

**Supplementary Table 1** Clinical characteristics of knee OA patients and traumatic amputees.

|  | **Knee OA patients** | **Traumatic amputees** | **χ^2^** | ***P* value** |
| --- | --- | --- | --- | --- |
| **Sex** |  |  | 0.00 | 1.000 |
| male | 10 (40.00%) | 4 (40.00%) |  |  |
| female | 15 (60.00%) | 6 (60.00%) |  |  |
| **Age range (years old)** |  |  | 0.08 | 0.960 |
| 50-59 | 10 (40.00%) | 4 (40.00%) |  |  |
| 60-69 | 9 (36.00%) | 4 (40.00%) |  |  |
| 70-79 | 6 (24.00%) | 2 (20.00%) |  |  |
| **Occupational types** |  |  | 1.57 | 0.905 |
| Government staff | 1 (4.00%) | 1 (10.00%) |  |  |
| Service personnel | 2 (8.00%) | 1 (10.00%) |  |  |
| Office staff | 6 (24.00%) | 3 (30.00%) |  |  |
| Housewife | 8 (32.00%) | 2 (20.00%) |  |  |
| Technician | 7 (28.00%) | 2 (20.00%) |  |  |
| Others | 1 (4.00%) | 1 (10.00%) |  |  |
